# Neuroanatomical and functional correlates in tic disorders and Tourette's syndrome: A narrative review

**DOI:** 10.1002/ibra.12177

**Published:** 2024-09-14

**Authors:** Anna Sara Liberati, Giulio Perrotta

**Affiliations:** ^1^ Division of Neuropsychology Faculty of Psychology, Università Telematica Internazionale “Uninettuno” Rome Italy; ^2^ Department of Psychological Sciences Forensic Science Academy Salerno Italy; ^3^ Division of Psychotherapy Institute for the Study of Psychotherapies (I.S.P.), Via San Martino Della Battaglia Rome Italy

**Keywords:** neuroanatomical correlates, tic, tic disorders, Tourette's syndrome

## Abstract

Tic disorders represent a developmental neuropsychiatric condition whose causes can be attributed to a variety of environmental, neurobiological, and genetic factors. From a neurophysiological perspective, the disorder has classically been associated with neurochemical imbalances (particularly dopamine and serotonin) and structural and functional alterations affecting, in particular, brain areas and circuits involved in the processing and coordination of movements: the basal ganglia, thalamus, motor cortical area, and cingulate cortex; however, more recent research is demonstrating the involvement of many more brain regions and neurotransmission systems than previously observed, such as the prefrontal cortex and cerebellum. In this paper, therefore, we summarize the evidence to date on these abnormalities with the intent to illustrate and clarify the main neuroanatomical differences between patients with tic disorders and healthy individuals.

## Graphical abstract

Tic disorders collectively represent a common neuropsychiatric condition in developmental age, the causes of which can be attributed to a variety of environmental, neurobiological, and genetic factors. From a neurophysiological point of view, the disorder has been classically associated with neurochemical imbalances (particularly dopamine and serotonin) and structural and functional changes affecting, in particular, the brain areas and circuits involved with the processing and coordination of movements.

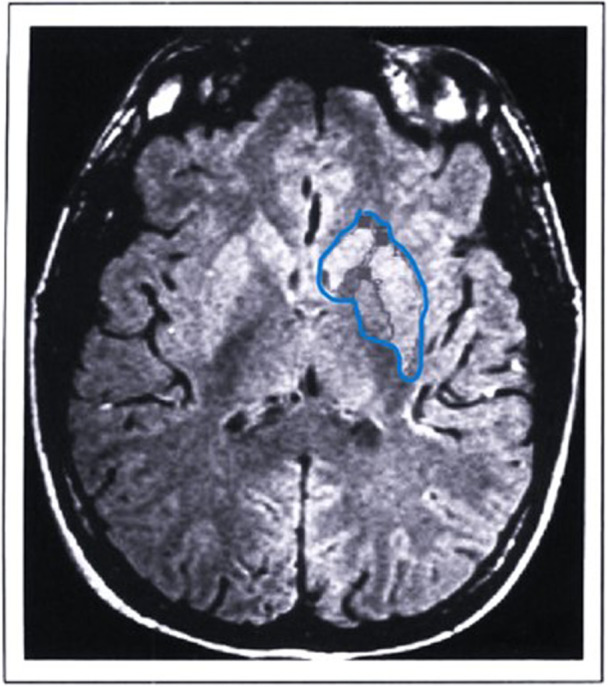

## 1 INTRODUCTION

Tics are defined as recurring, impulsive, and sudden movements, sounds, or vocalizations, sometimes bizarre, carried out without an apparent purpose, which generally appear in childhood and then regress in adulthood, although in more severe cases they can persist throughout the individual's life. The estimated incidence is five cases per 1000, with a male/female ratio of about 3:1.^1^ Symptoms are exacerbated by the presence of anxious states, excitement, and fatigue and tend to fade in moments of calm or during the execution of engaging and cognitively demanding activities. Although they differ in type, severity, characteristics (they can indeed be simple, complex, or compulsive), and course, these disorders tend to have typical characteristics and manifest in a similar way among affected individuals; therefore, the diagnostic macro‐category includes within it four sub‐forms, conventionally listed in a hierarchical order of decreasing severity: Gilles de La Tourette syndrome, chronic (or persistent) tic disorder, transient tic disorder, and tic disorders without (or with other) specification, characterized as follows:

1. *Tourette's Syndrome (TS) (or Gilles de la Tourette Syndrome)*. It is characterized by the presence of multiple motor tics, as well as one or more vocal tics, although not necessarily in conjunction with each other. Tics can have symptomatic fluctuations in frequency but must persist for over 12 months from onset, which generally occurs before the age of 18. Symptoms must not be attributable to the physiological effects caused by the use of substances or drugs, nor to another pathophysiological condition.
2. *Chronic Motor or Vocal Tic Disorder*. It is characterized by the manifestation of motor or vocal tics, single or multiple (never both), sometimes discontinuous over time but present for more than a year from the onset of the first tic episode (occurred before the age of 18). In this case too, it is necessary to exclude that the disorder derives from the effects of a substance or a medical condition.
3. *Transient Tic Disorder*. Characterized by motor and/or vocal tics, single or multiple, present for less than a year compared to the onset of the first tic (before the age of 18), not attributable to the psychophysiological effects due to substance or drug abuse, nor to other medical conditions.
4. *Tic Disorders Without (or With Other) Specification*. This category includes those cases in which, although the patient may manifest the characteristic symptoms of a tic disorder, with onset, duration, and impairment of quality of life relatively similar to those observable in typical cases, these elements do not satisfy, or do not fully meet, the criteria established for the specific diagnosis.

Therefore, to make a correct diagnosis, it is important to exclude that the symptoms may derive, in addition to the use of drugs or other substances, from more purely neurological diseases, such as myoclonus, Huntington's chorea, or restless legs syndrome, or from other neuropsychological disorders, such as attention deficit/hyperactivity disorder (ADHD) and obsessive‐compulsive disorder (OCD). Therefore, it is always necessary to evaluate the presence or absence of certain characteristics, including:

1. The patient's ability to voluntarily exercise inhibitory control over the enactment of the tic.^2^
2. Presence of “premonitory impulses.” That is, unstructured sensations, perceptions, or mental experiences resulting from an increase in internal tension that precedes and finds subsequent relief in the expression of the tic.^3^
3. Variability. Tics can vary in duration, frequency, intensity, and location of the motor or vocal act, thus clearly differentiating themselves from the purely neurological stereotypies observed in diseases, for example, Parkinson's disease or chorea.^3^

Despite the proven genetic, perinatal, and immunological components of these disorders, the evidence available to date demonstrates a certainly multifactorial origin of the same, complicating the investigation and understanding of the neurophysiological anomalies that characterize them. In recent years, however, the presence of functional and/or structural modifications affecting multiple cortical and subcortical structures, in particular the basal ganglia and related neuromodulatory circuits, has been well documented in these patients. This complex neural wiring indeed involves several structures whose concerted activity processes and coordinates mainly motor inputs but also emotional and cognitive inputs related to these.^4^, ^5^ Due to the small number of subjects examined in these studies, however, it is very difficult to obtain consistent, exhaustive, and homogeneous results, especially when outcomes related to their heterogeneity, different manifestation and persistence of symptoms, different diseases (when present) in comorbidity, and specific type of pharmacological and therapeutic treatment (if prescribed) are analyzed. Therefore, a sufficiently complete neurobiological model is still lacking, capable of explaining the neural dynamics underlying the manifestation, as well as the inhibition of tics. Starting from this need, the objective we have set ourselves with this work is to offer an examination of the main evidence currently available in the literature on the structural and functional anomalies of the circuits and brain structures involved, in particular, with TS (as this morbid condition appears in the literature to be more studied), and to compare them with normal neural functioning, to obtain a clearer picture of the neurobiological correlates of this disorder.

**Figure 1:**
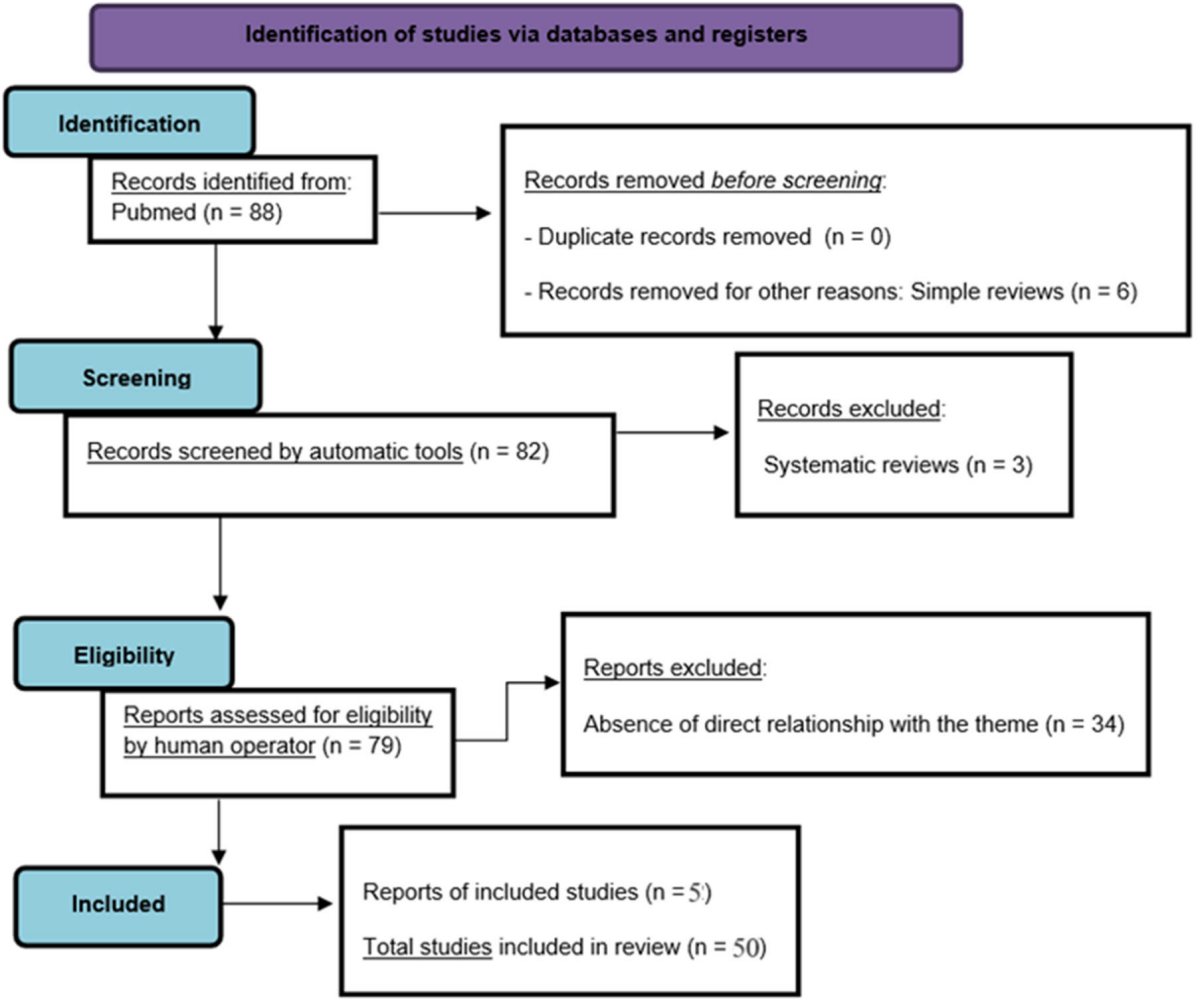
The preferred reporting items for systematic reviews and meta‐analyses (PRISMA) flow diagram 84 template. [Color figure can be viewed at wileyonlinelibrary.com]

The authors searched PubMed, from January 1998 to January 2024, using “tics” and “neural correlates,” selecting 45 eligibility results. Five more references were added, for a total of 50 results. The search was not limited to English‐language papers (Figure 1).

## 2 NEUROANATOMICAL AND FUNCTIONAL CORRELATES OF TS

The most severe type of tic disorder is TS, a neurodevelopmental disorder characterized by multiple motor tics, simple and/or complex, and one or more phonic tics—not necessarily present simultaneously—lasting at least 1 year, generally arising during childhood or adolescence, but capable in some cases of persisting even into adulthood.^4^ Tendentially, motor symptoms start before phonic ones and often patients report experiencing some sensations anticipating the tic, such as itching, tingling, or increasing tension states. Although most adult patients are able, in certain circumstances, to voluntarily restrain their tics, the condition can significantly interfere with the quality of life and the functioning of the person, making communication, the performance of normal daily activities, and, not least, social relationships difficult.^5^ Furthermore, comorbidity with other cognitive and/or behavioral disorders, including mood and anxiety disorders, OCD, and ADHD, is common.^6^, ^7^

The specific causes of this pathology, as well as other tic disorders, are still under investigation, but the most accredited hypothesis believes that they emerge from a heterogeneous combination, differing from subject to subject, of environmental, psychological, genetic, immunological, and neurobiological factors. The latter are particularly associated with imbalances in dopaminergic and serotonergic neurotransmission and with structural and functional anomalies located in various brain areas, such as those responsible for the processing and production of language and those cortico‐striatal ones that modulate the planning and motor output, connecting the frontal, motor cortical, and sensorimotor areas to the striatum.^8^, ^9^

However, recent advances in neuroimaging technologies are demonstrating the involvement of other brain areas, in addition to those classically recognized, such as the parietal, temporal, occipital cortical regions, and the cerebellum,^10^ as well as dysfunctions in the GABAergic, glutamatergic,^11^ cholinergic, and even endocannabinoid systems.^12^

### 2.1 Basal ganglia

**Figure 2:**
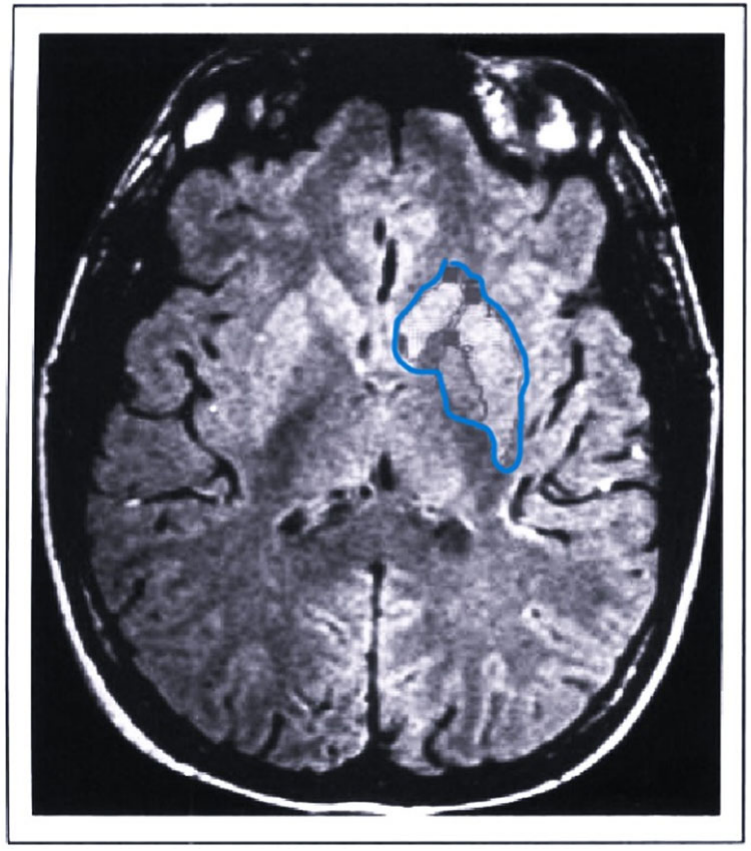
Two‐dimensional axial MR image, with spatial identification of the basal ganglia, circumscribed with the blue contour lineo.^19^ [Color figure can be viewed at wileyonlinelibrary.com]

Basal ganglia (BG) refers to the set of monoaminergic nuclei and nerve circuits belonging to the areas of the basal forebrain and midbrain, mainly involved in the management and planning of voluntary movement. These include the striatum (in turn composed of the nuclei: caudate, putamen—which together form the lenticular nucleus—and accumbens), the globus pallidus (divided into an external, lateral portion and an internal, medial one), the subthalamic nucleus, the thalamus, and the substantia nigra. These structures, as a whole, act by integrating motor and/or cognitive inputs from the cortex and projected to the striatum, which in turn conveys them to the thalamus so that they are returned again to the cortex, for the planning, intention, and/or execution of the response movement. In particular, two paths are known, opposite but complementary to each other, through which the motor circuit is completed: a so‐called “direct” path, excitatory, which reaches the thalamus from the striatum passing through the internal portion of the globus pallidus, and an “indirect” path, inhibitory, which also involves the subthalamus. The balance between these two loops is fundamental for the correct organization and management of movements. Although with sometimes contrasting results, there is much scientific evidence that demonstrates the implication of the BG in the pathophysiology of TS. In most research, a reduction in the functionality and volume of these regions has been detected, associated with a dysfunction of the striatal GABAergic networks (which, in turn, leads to an excessive release of dopamine, from which the tic behavior derives), supporting the hypothesis about the existence, in these patients, of an impairment of the functions responsible for motor control.^13^ However, the specific mechanisms underlying this dysfunction are still under investigation today, also given the absence of clear data regarding the characterization of the specific cellular and subcellular species involved in them.^4^ Various functional magnetic resonance imaging (fMRI) studies have nevertheless highlighted the presence, in subjects with TS, of morphological alterations affecting, in particular, the striatal caudate nucleus,^14^ whose volume has also been inversely correlated with the severity of the tics manifested in early adulthood. For example, Peterson et al.^15^ documented a decrease in the volume of the BG in a cohort composed of over 150 patients, both adults and children, with TS. In children, the reduction in the volume of the caudate was particularly observed, while in adult subjects the volumetric reductions were more widespread, affecting not only the caudate but almost all the structures of the BG. This evidence is associated with those previously detected by a study conducted on twins affected by TS^16^ in which it was observed that, in each pair, the most severely affected brothers showed a significantly smaller right caudate compared to the twin with less important symptomatology. However, it remains to be clarified whether these anomalies precede or follow the development of TS. On the contrary, however, other studies^17^ have not found any anomaly in the volume of the caudate in any of the subjects analyzed, while a reduction in the dimensions of the putamen, bilaterally, has been documented. As for the specifically vocal tics—verbal and nonverbal—such as, for example, continuously clearing the throat, grunting, cursing (coprolalia), the repetition of terms and sounds (echolalia), and so forth, despite the absence of certain data regarding the mechanisms and networks involved in their emission, a central role of the nucleus accumbens (NAc) and the fibers that connect it with the limbic system is hypothesized, as demonstrated in some studies conducted on nonhuman primates.^18^ In fact, by hyperstimulating the NAc through microinjections of Bicuculline (a GABA antagonist), researchers have evoked complex repetitive vocalizations in all the subjects examined, supporting the hypothesis that vocal tics in TS may derive from a dysfunction of the cortico‐subcortical GABAergic circuits (Figure 2).

### 2.2 Thalamus

Thalamus, as a paired structure, located bilaterally at the edges of the third ventricle, constitutes the “collection and sorting center”^20^ for sensitive and motor connections from—and to—the cerebral cortex. Although it is an integral and fundamental part of the cortico‐striato‐thalamo‐cortical circuit (CSTC), whose role in the genesis of tic symptoms and their associated compensatory responses is relatively well known, we believe that the thalamus deserves separate treatment. Studies conducted through fMRI^21^ have indeed highlighted a specific hyperactivation of the thalamus during the voluntary suppression of tic symptoms, which would seem to be independent, and yet correlated, with variations in the activity of the structures composing the BG, suggesting the need for a concerted action of these structures for the inhibition of the tic. Furthermore, it has been observed how the severity of the symptoms manifested by the patients is more closely correlated with the extent of the variation in activity of the thalamic and subcortical regions, compared to that of the cortical regions,^21^ underlining the centrality of this structure in tic disorders. Other research has also documented how the presence of lesions or anomalies, or the clinical application of inhibitory stimulation protocols in the subthalamic regions, can reduce the expression of tics,^22^ while, on the contrary, lesions that directly affect the thalamus would seem to intensify them.^23^ These and other results have led to the hypothesis that a dysregulation of thalamo‐cortical activity may be the key cause of the symptomatic manifestation of tic syndromes. As for the morphovolumetric characteristics of the thalamus, there are somewhat contrasting results in the literature. For example, one study^24^ highlighted an increase in the size of the left thalamus in a sample of 18 male adolescents with TS, who had never received clinical or pharmacological treatments. Another study,^25^ conducted on 15 adults who had never followed a pharmacological protocol, found no noteworthy volumetric anomaly. And finally, a third work,^26^ in which, however, the treatment (if followed) by the subjects was not specified, documented a reduction in thalamic volumes in a sample of children and preadolescents with a diagnosis of TS. More recent research^27^ seems to confirm the observations of the first of these studies, having actually found, in a more heterogeneous and larger sample of subjects with TS, an increase, compared to the norm, in the size of the thalamic nuclei, particularly on the left, not attributable to any comorbid diseases or the use of drugs and/or treatments. However, it must be said that the different clinical and pharmacological histories, as well as the age, sex, and sample size of subjects analyzed in the various works, together with the significant differences in the image processing techniques used, do not allow for an adequate explanation and interpretation of the divergences between the results obtained, suggesting the need for more in‐depth investigations.

### 2.3 Prefrontal cortex (PFC)

Although not always with converging results, most of the fMRI investigations available in the literature to date have detected the presence of various volumetric alterations in the fronto‐cortical regions in patients with TS. In particular, an increase in the size of the dorsal prefrontal and parieto‐occipital areas has been observed, both in adults and children (more evident in male children), and, on the contrary, a reduction of the lower occipital and premotor regions (more evident in female children and adults of both sexes).^28^ It is believed that these differences, correlated with the sex and age of the patients, are attributable to various factors, including the stages of brain development, the action of sex hormones, the severity of symptoms, plastic changes dependent on long‐term activity (presumably of an adaptive or compensatory nature) resulting from the persistence of tics even in adulthood, as well as the influence of the socio‐cultural environment of reference and the different coping strategies and responses to stress.^29^ Furthermore, since lower orbitofrontal and parieto‐occipital volumes have often been associated with more serious symptomatic manifestations, the most common hypothesis is that patients who show a reduction in the activity of these areas also have less ability to control the emission of these unwanted behaviors. Consistent with this, numerous preclinical and clinical studies have suggested that the orbitofrontal PFC may play an important role especially in the inhibitory control of tics.^30^, ^31^ To support this theory, there is also the observation that when patients with TS voluntarily suppress the emission of tics, the activation of the PFC (detectable in particular at the level of the lower frontal gyrus) is generally accompanied by the simultaneous reduction of BG activity.^32^ These results, as a whole, further support the hypothesis of the primary involvement of the CSTC—responsible for the management of habitual motor behavior, including the transition from the selection of the action directed to the objective, to compulsive action—in the generation of tics.^33^

### 2.4 Motor cortical areas (MCA)

By virtue of their primarily motor nature, the MCA, which includes the primary motor cortex (MC1), the premotor cortex (PMC), the supplementary motor area (SMA), and the superior parietal cortex (SPC), undoubtedly play a crucial role, both in the manifestation of tics and in their severity. Zapparoli et al.^34^ for example, found that in patients with TS there is an imbalance in the connectivity between these areas and their reference circuits (including the CSTC), directly correlated with the severity of the symptoms they manifest, measurable with tools such as the *Yale Global Tic Severity Scale* (YGTSS). The greater the severity of the syndrome, the more significant this imbalance turned out to be. Thus, a higher connectivity between the MC1 and the structures belonging to the CSTC would correspond to a greater severity of motor symptoms, and therefore of tics; while, on the contrary, the prevalence of connections between the MC1 and the PMC was associated with milder symptomatology. However, at the current state, it is still quite complicated to accurately characterize the motor substrate of tics, mainly due to movement artifacts and frequent comorbidity with compulsive disorders, as well as the subjective, physiological, and psychological characteristics of individual patients. For example, in the classic preimpulse inhibition paradigms, aimed at investigating the ability to inhibit automatic or impulsive motor responses, children and adults with TS generally show alterations in motor neural activity of a diffuse type, suggesting the existence of mostly nonspecific dysfunctions of somatosensory gating.^35^ In any case, in line with what has already been described previously, several studies have well documented the presence, in these patients, of dysfunctions in the neurotransmission of GABA, also affecting the motor areas, in particular, the MC1.^36^

### 2.5 Cingulate cortex (CC)

**Figure 3:**
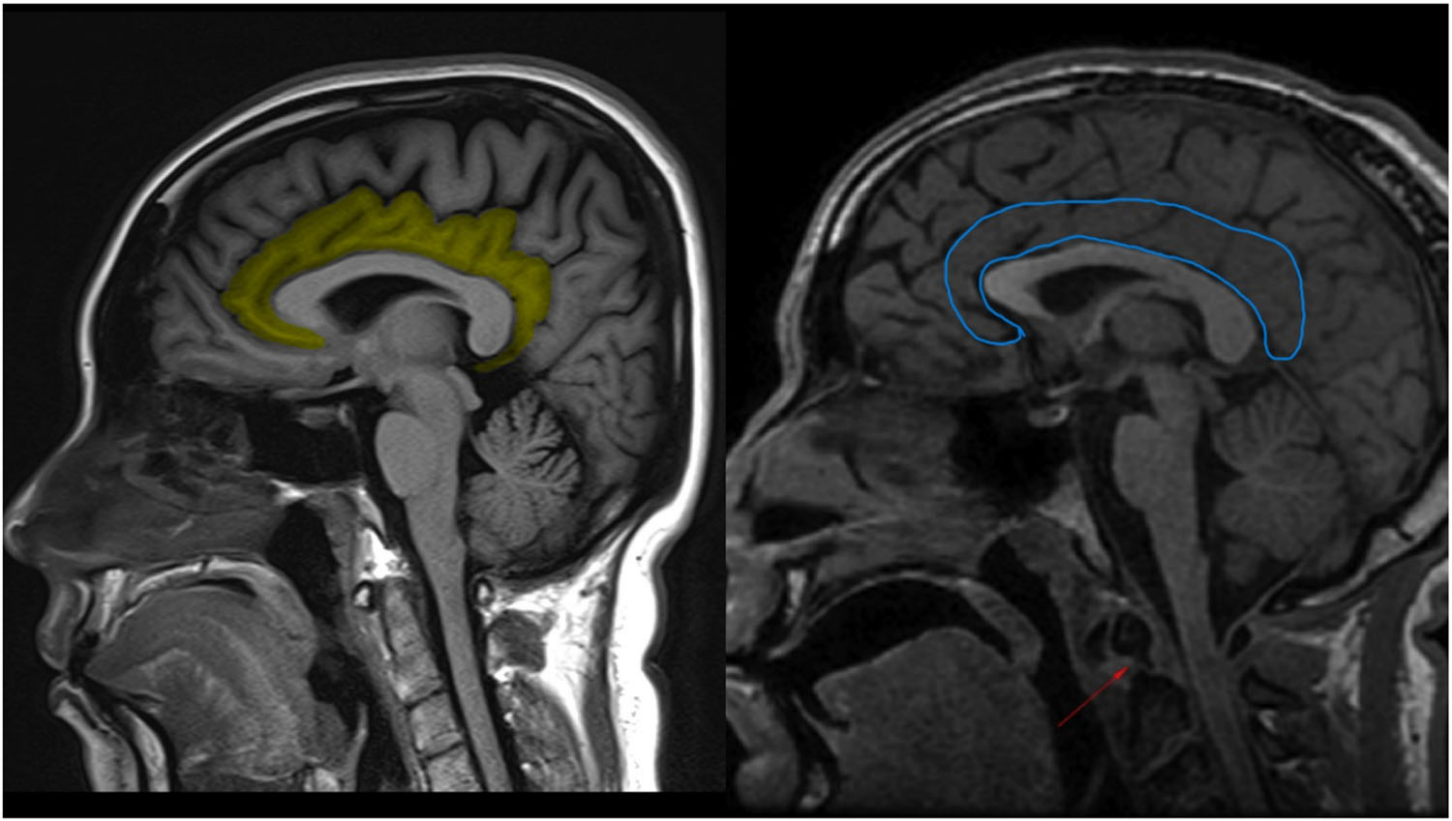
Identification and comparison between CC of a healthy subject (Left) and CC of a subject with TS (Right). Case courtesy of Bruno Di Muzio, Radiopaedia.org, rID: 35221. 3B (R).^43^ [Color figure can be viewed at wileyonlinelibrary.com]

CC, also known as the “cingulate gyrus,” extends from the subcallosal gyrus in the frontal lobe, anteriorly, to the isthmus, posteriorly. It results in the superior convexity of the corpus callosum, which the gyrus entirely surrounds and from which it is separated by the callosal sulcus.^37^ The CC can ideally be distinguished into three main subregions: anterior (ACC), located inferiorly to the superior frontal gyrus and underlying the rostrum of the corpus callosum; median (MCC), located below the paracentral lobule; and posterior (PCC), underlying the precuneus. Each of these areas presents a distinct cytoarchitecture and specific connections with other brain districts, accounting for their respective specific functionalities. The ACC establishes numerous interconnections with the frontal cortex (FC) and PFC, the CSTC, and various limbic structures, assuming, among its numerous functions, also an important role in the emotional processing of language, movement, initiation, and motivation of a targeted behavior. The MCC sends and receives projections to the dorsolateral prefrontal cortex (DLPFC), the SMA, the parietal cortex, and the spinal cord, and is involved in decision‐making mechanisms, topokinetic memory, and voluntary motor control. Finally, the PCC communicates with the parietal and temporal lobes, the hippocampus and the entorhinal cortex, and the anterior thalamic nucleus; therefore, it participates in the management and processing of memory and spatial orientation, associative learning, movement, and sensorimotor stimuli.^38^ MRI investigations conducted on various samples of patients with TS^39^ have shown the presence of a cortical thinning and a volume lower than the norm affecting both the ACC and the MCC and, on the contrary, an increase in thickness in the PCC. It is assumed that these alterations may reflect the inadequate inhibition of local neural transmission by striatal GABAergic neurotransmitters, a hypothesis also supported by some magnetic resonance spectroscopy (MRS)^40^ and positron emission tomography (PET)^41^ studies. Furthermore, the fractional anisotropy (FA) and the apparent diffusion coefficient (ADC) measured by diffusion tensor imaging (DTI) demonstrate a volumetric reduction also affecting the white matter surrounding the CC.^42^ Studies, instead, of resting‐state functional magnetic resonance imaging (rsfMRI) have identified anomalies in the functional connectivity between ACC and PCC and the internal globus pallidus, which were correlated with the specific severity and complexity of the tics manifested by the individual subjects examined^31^ (Figure 3).

### 2.6 Cerebellum

**Figure 4:**
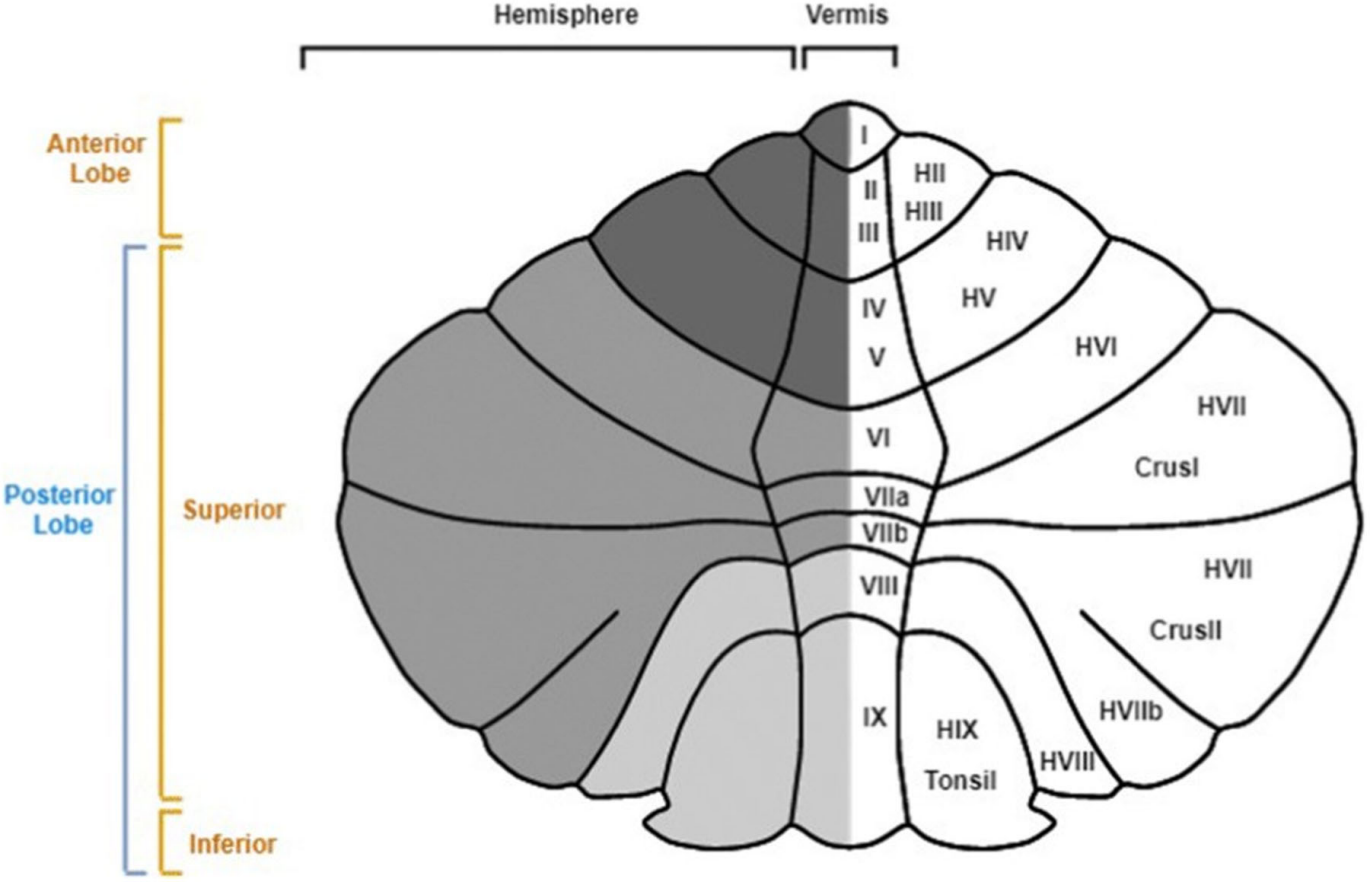
Cerebral cortical activity following noninvasive cerebellar stimulation. The anatomical image is detailed by sections and subsections to distinguish areas compromised by TS.^47^ [Color figure can be viewed at wileyonlinelibrary.com]

The cerebellum is traditionally associated with predominantly motor functions. Lesions in this area (especially in the anterior lobe) can indeed lead to alterations in the temporal and spatial coordination of movements, hypotonia, balance and posture deficits, motor learning impairments, and so forth. However, in recent years, various research are revealing its importance also in relation to higher cognitive abilities and behavior. Some neural circuits have been discovered, for example, the cortico‐ponto‐cerebellar (CPC) circuit and the cerebello‐thalamo‐cortical (CTC) circuit, that connect the cerebellum with the frontal, temporal, and parietal cortices and with the paralimbic regions, suggesting its involvement with cognitive, emotional, linguistic, and even visuo‐spatial functions.^44^ Starting from these observations, recent evidence seems to demonstrate an involvement of the cerebellum also in the genesis of tics, especially with regard to the CTC.^45^ It has indeed been observed how the increase in striatal dopamine, induced by the dysfunction of the GABAergic networks in the BG, resulting in a focal abnormal excitation in the striatum, is able to also influence cerebellar activity, triggering a negative feedback mechanism—from the cerebellum to the BG—from which would derive the disinhibition of the descending motor pathways at the base of the generation of tics. Furthermore, structural imaging studies have shown alterations in the gray matter (GM) of the cerebellum in patients with TS, with differences related to the presence or absence of comorbidity. In fact, in subjects with TS associated with OCD, ADHD, or autism spectrum disorder (ASD), a hypotrophy of the cerebellar lobule, subregion “Crus I” (area involved in particular with higher order cognitive functions),^19^ was observed; while in patients with TS without other associated conditions, a reduction in GM was found always in the cerebellar lobule, but at the level of the subregion “VIIIa” (mainly involved with sensorimotor processing),^46^ suggesting the need to investigate more deeply the neuroanatomical characteristics and the potential contribution of the cerebellum in the symptomatology and pathophysiology of TS and other tic disorders (Figure 4).

In conclusion, the development of TS may involve a series of neuroanatomical areas, including BG, thalamus, PFC, and cerebellum. Below is a summary table of the neuroanatomical differences between a subject with TS and a healthy one (Table 1).

**Table 1:** Neuroanatomical and functional differences between an average healthy subject and a subject with TS.

Neuroanatomical areas	Healthy subject (average young adult)	Person with TS
*Basal Ganglia (BG)*	Set of nuclei and monoaminergic nervous circuits belonging to the areas of the basal forebrain and midbrain, mainly involved in the management and planning of voluntary movement. As a whole, these structures act by integrating the motor and/or cognitive inputs coming from the cortex, projected to the striatum, and then conveyed to the thalamus, from which are returned back to the cortex for planning, intention, and/or or the execution of the response output.	Presence of widespread morphological and functional alterations, most evident in the striatal caudate nucleus and putamen, associated with a dysfunction of the GABAergic networks, resulting in an excessive release of dopamine, responsible for generating motor tic behavior. However, for what concerns the vocal tics, the structure most affected by volumetric alterations is the nucleus accumbens (NAc), once again attributable to dysfunctions of the cortico‐subcortical GABAergic circuits.
*Thalamus*	Paired gray matter structure, composed of approximately 60 nuclei, nestled in the dorsal part of the diencephalon, deep into the cerebral cortices. It acts as the “brain's central hub,” relaying and integrating impulses between the higher centers of the brain and the peripheries, and absolving several important functions, such as sensory and motor elaboration, cognition, attention, memory, speech, and emotion.	In patients with TS, a specific hyperactivation of the thalamus is generally found during the voluntary suppression of tic symptoms, independent of, and yet correlated with, variations in BG activity. However, although morphostructural anomalies affecting the thalamus are documented, these appear to vary (increases or decreases in volumes) based on the type of patient, the severity of the symptoms, the treatment followed, and the presence or absence of comorbidities.
*Prefrontal Cortex (PFC)*	Located anteriorly to the frontal lobe, the PFC—one of the last regions of the cortex (neocortex) to develop—plays a pivotal role in executive function, orchestrating, together with other areas with which it is reciprocally connected, a complex symphony of motor, cognitive, and emotional functions.	In patients with TS, both adults and minors, the following are observed: an increase in the size of the dorsal prefrontal and parieto‐occipital areas—more evident in male children—and a volumetric reduction of the lower occipital and premotor regions—more evident in girls and in adults of both sexes.
*Cerebellum*	Located in the posterior cranial fossa behind the pons and medulla oblongata, separated from them by the fourth ventricle. It is divided into two hemispheres (left and right) and three lobes (anterior, posterior, and flocculonodular). It is responsible for dealing with motor learning, coordination, and precision of motor functions, but it also plays a role in cognitive, emotional, linguistic, and visuospatial functions, thanks to connections [cortico‐ponto‐cerebellar (CPC) and the circuit cerebello‐thalamo‐cortical (CTC)] with the frontal, temporal, parietal cortices and paralimbic regions.	Structural imaging studies have highlighted alterations of the cerebellar GM in patients with TS, with differences relating to the presence or absence of comorbidities. In TS associated with OCD, ADHD, or ASD, hypotrophy of the cerebellar lobule, subregion “Crus I” (higher order cognitive functions) is found; while in TS without other associated conditions, a reduction of the GM is observed at the level of subregion “VIIIa” (sensorimotor processing).
*Motor Cortical Areas (MCA)*	Subdivided into: primary motor cortex (MC1): located on the precentral gyrus and the anterior paracentral lobule in the frontal lobe. Responsible for the planning, control, and execution of voluntary movements. Premotor cortex (PMC): immediately anterior to the primary motor cortex (Brodmann area 6). Particularly involved in preparation for movement, particularly in the proximal muscles. Supplementary motor area (SMA): located on the medial surface of the longitudinal fissure. Involved in the stabilization and postural coordination of the body. Superior parietal cortex (SPC): located within the parietal lobe. Although it is not strictly a motor area, it plays a fundamental role in various functions related to perception, attention, and spatial awareness. Overall, these cortical areas work together to orchestrate voluntary movements, integrate sensory information, and maintain spatial awareness.	In patients with TS there is an imbalance in the connectivity between these areas and their reference circuits (including the CSTC), directly correlated to the severity of the manifested symptoms: the greater the severity of the syndrome, the more important the imbalance. Therefore, a higher connectivity between MC1 and the structures belonging to the CSTC corresponds to a greater severity of motor symptoms, while the prevalence of connections between MC1 and PMC is associated with milder symptoms.
*Anterior cingulate cortex (ACC) and Medial cingulate cortex (MCC)*	ACC is the most distal portion of the cingulate gyrus (bilateral structure surrounding the corpus callosum). Given the direct connections, it establishes with the PFC and some limbic structures (amygdala, hypothalamus, and hippocampus), it participates in the encoding of emotions, particularly anxiety, anger, and fear, regulates some endocrine and vegetative functions, and participates in emotional language production. MCC sends and receives projections to the DLPFC, SMA, parietal cortex, and spinal cord, and is implicated in decision‐making mechanisms, topokinetic memory, and voluntary motor control.	Neuroimaging studies have demonstrated, in TS patients, the presence of cortical thinning and smaller‐than‐normal volumes of both the ACC and the MCC.
*Posterior cingulate cortex (PCC)*	The caudal portion of the cingulate gyrus. Being an integral part of the “Papez circuit,” it plays an important role in memory consolidation and recall, but also in associative learning, regulation of social behavior, and some higher cognitive processes.	In patients with TS, an increase in thickness in the PCC is also found, associated with a general volumetric reduction of the surrounding white matter. While rsfMRI studies have identified anomalies in the functional connectivity between the ACC, PCC, and globus pallidus internus, correlated to the specific severity and complexity of the tics manifested by the individual subjects examined.

## 3 CONCLUSIONS AND FUTURE PERSPECTIVES

Tic disorders, particularly TS, present a complex and multifaceted clinical picture. The manifestation of these disorders varies significantly based on individual characteristics, age, sex, and the presence or absence of comorbid conditions. The pathophysiology of these disorders is believed to involve a combination of genetic, environmental, psychological, immunological, and neurobiological factors.^48^, ^49^

Neuroimaging studies have revealed structural and functional anomalies in various brain areas, including the BG, thalamus, CC, and cerebellum. These findings suggest that the CSTC circuit, which is responsible for managing habitual motor behavior, plays a crucial role in the generation of tics.^50^ However, the specific mechanisms underlying these dysfunctions remain under investigation. The heterogeneity of the patient population, the varying severity and persistence of symptoms, the presence of comorbid conditions, and the specific types of treatments followed by patients make it challenging to draw definitive conclusions about the pathophysiology of these disorders and their neuroanatomical correlates.

Future research should focus on well‐defined patient samples and employ rigorous inclusion and exclusion criteria. Longitudinal studies, which track the same patients from childhood to adulthood, could provide valuable insights into the development and symptomatic evolution of these disorders and their neuroanatomical changes over time. Furthermore, a deeper understanding of the characteristics of premonitory impulses and the mechanisms underlying tic inhibition could shed light on the neural correlates of these disorders. This knowledge could potentially lead to the development of more targeted and effective treatments for TS and other tic disorders.

## AUTHOR CONTRIBUTIONS

Giulio Perrotta conceived the approach and supervised. Anna Sara Liberati wrote the paper. All authors have read and approved the final manuscript.

## CONFLICT OF INTEREST STATEMENT

The authors declare no conflicts of interest.

## ETHICS STATEMENT

Not applicable.

## TRANSPARENCY STATEMENT

Authors affirms that this manuscript is an honest, accurate, and transparent account of the study being reported; that no important aspects of the study have been omitted; and that any discrepancies from the study as planned (and, if relevant, registered) have been explained.

## Data Availability

Not applicable as no new data are generated in this review article.
